# Increased rates of proton pump inhibitor deprescription: a retrospective cohort of patients with upper gastrointestinal bleeding requiring endoscopic intervention

**DOI:** 10.1093/jcag/gwaf021

**Published:** 2025-09-24

**Authors:** Kevin Kecskemeti, Mark Borgaonkar, Jerry McGrath

**Affiliations:** Department of Medicine, Temerty Faculty of Medicine, University of Toronto, Toronto, ON, M5S 3K3, Canada; Department of Gastroenterology, Faculty of Medicine, Memorial University of Newfoundland, 300 Prince Philip Drive St. John's, NL, A1B 3V6, Canada; Department of Gastroenterology, Faculty of Medicine, Memorial University of Newfoundland, 300 Prince Philip Drive St. John's, NL, A1B 3V6, Canada

**Keywords:** proton pump inhibitor, ulcer disease, deprescribing, upper GI bleeding

## Abstract

**Objective:**

Proton pump inhibitors (PPIs) are widely prescribed but inappropriate indications and concerns over long-term side effects have led to recommendations to deprescribe PPIs in certain patients. We previously found a 4-fold increase in PPI deprescription in patients with esophageal strictures. This study aims to assess the PPI deprescription rate in patients with upper gastrointestinal bleeding (UGIB).

**Methods:**

All patients from 2 gastroenterology practices who received endoscopic treatment for UGIB during the years of 2015-2022 were identified using physician billing codes. We defined PPI deprescription as either a 50% dose reduction, frequency reduction, or complete medication discontinuation at the time of endoscopic intervention compared to the established PPI therapy from the 3 months prior. We compared the rate of PPI deprescription between 2 time periods 2015-2018 (group 1) and 2019-2022 (group 2).

**Results:**

Three hundred one UGIB managed with endoscopy were analyzed. Patients in group 2 had a significantly higher rate of PPI deprescription than group 1 (15% vs 4%; *P *< .002). Patients with peptic ulcer disease (PUD) had a significantly higher PPI deprescription during the second time period (16% vs 0%; *P *= .028). Among patients with repeat UGIB, 10% had their PPI deprescribed.

**Conclusions:**

Proton pump inhibitor deprescription in patients with UGIB treated with endoscopic intervention was more common in the second time period. This corresponds to when PPI deprescription guidelines were distributed. Physicians should ensure the appropriate application of PPI deprescription guidelines and continuation of PPI therapy for patients with strong indications.

## Introduction

Proton pump inhibitors (PPIs) have become one of the most widely prescribed medications to treat reflux disorder, dyspepsia, and peptic ulcer disease (PUD) among many indications. A recent systematic review estimates that one-quarter of all adults globally use a PPI.^1^ In a recent Canadian study, PPI usage in the province of Alberta was estimated at 12% of the general population.^2^ Alongside this rising trend in PPI usage, there have been concerns raised about increased risk of polypharmacy, nutrient malabsorption,^3^ enteric infections,^4^ and dementia^5^ that were identified in observational studies. This has led to recommendations to reduce and deprescribe proton-pump inhibitors in low-medium risk patients with upper gastrointestinal disorders or those with unclear indications. One of the popular resources that was widely published is the PPI deprescription toolkit by Choosing Wisely Canada.^6^ Assessing these trends was the focus of our previous study, where we analyzed the frequency of PPI deprescription in patients requiring endoscopic therapy for esophageal strictures.^7^ We found that there was a 4-fold increase in PPI deprescription in recent years with the majority being classified as inappropriate.^7^ As such, we wanted to expand the scope of our research to investigate PPI deprescription in another high-risk patient population—those who experienced upper gastrointestinal bleeding (UGIB).

## Methods

This retrospective cohort study analyzed patients who received endoscopic treatment for UGIB between the years of 2015-2018 (group 1) and 2019-2022 (group 2). These dates were selected as they coincided with PPI deprescription guidelines released in March 2018 encouraging PPI deprescription in low-to-medium risk patients with upper gastrointestinal disorders.^6^ Our study aimed to analyze PPI deprescription before and after these guidelines were released. The project received Memorial University Health Research Ethics Board approval on February 21, 2023.

All patients from 2 academic gastroenterology practices who received endoscopic treatment for UGIB during the study years were included in the study. These patients were identified using physician billing codes. To reduce bias the authors opted to select all patients from multiple practices as opposed to using a single practice. Once the patients were identified we conducted a chart review from the electronic medical database. We analyzed endoscopy reports, narrative reports, medication forms for the following variables: sex, age, date of endoscopic treatment, and diagnosis after endoscopy, previous PPI medication dose, and frequency. All the patients were coded using a standardized data sheet and entered into SPSS for data analysis.

For the purpose of the study, we defined PPI deprescription as either a 50% dose reduction, frequency reduction, or complete medication discontinuation at the time of endoscopic intervention compared to the baseline PPI regimen from the preceding 3 months, as documented in the patient’s medical records.

We documented and analyzed the patient’s first endoscopic intervention for a UGIB. If a patient had a repeat UGIB within 3 months of their first endoscopic intervention, only the first procedure was included to avoid duplicate analysis of research subjects.

For our sample size calculation, we estimated a deprescription rate of 5% for group 1 and 25% for group 2. These values were influenced based on the findings from our prior study. Therefore, using an alpha value of .05 and a beta value of . 20, we required a sample size of at least 47 patients per group to achieve statistically significant results.

## Results

### Inclusion criteria

In total we identified 349 UGIB treated with endoscopic intervention between the years of 2015-2022. Nine procedures were excluded due to incomplete medical records such as missing endoscopy reports. Fifteen procedures were excluded as they were not for an active UGIB. These included variceal banding for nonactive bleeds or polypectomies. Twenty-four procedures were excluded from the analysis as they were repeat UGIB interventions occurring within 3 months of a first endoscopic intervention which was already captured in the study. In total, 301 UGIB treated endoscopically from 249 unique patients were included in the analysis.

### PPI demographics and characteristics

Demographic data were collected from the 301 UGIB and 249 unique patients. The average age of the cohort was 66.4 ± 13.9. The sample consisted of 164 males (66%) and 85 females (34%). Among the 249 unique patients, the most common diagnoses following endoscopic treatment were PUD, gastric antral vascular ectasia (GAVE), and angiodysplasia which accounted for 67.5% of the cohort (Table 1). This analysis was repeated for all 301 endoscopic procedures, which demonstrated similar proportions of diagnoses, with PUD, GAVE, and angiodysplasia remaining the most common. The proportion of patients with GAVE was higher when analyzing all endoscopic procedures. This is explained by patients undergoing multiple endoscopic treatments for GAVE during the study years (Table 2).

**Table 1.** Endoscopic diagnosis following treatment of upper gastrointestinal bleeding among the 249 unique patients.

Diagnosis	Number of patients	Percentage of sample
**Peptic ulcer disease**	88	35.3
**GAVE**	44	17.7
**Angiodysplasia**	36	14.5
**Esophageal varices**	34	13.7
**Gastritis**	10	4.0
**Dieulafoy lesion**	7	2.8
**Esophageal ulcer**	5	2.0
**Esophagitis**	5	2.0
**Mallory-Weiss tear**	3	1.2
**Carcinoma**	2	0.8
**Gastric varices**	2	0.8
**Other** ^a^	13	5.2
**Total**	249	100.0

Abbreviation: GAVE, Gastric antral vascular ectasia.

a Other includes: Gastrojejunostomy bleed, heparin-induced thrombocytopenia, and not specified.

**Table 2.** Endoscopic diagnosis following treatment of upper ­gastrointestinal bleeding among the 301 procedures.

Diagnosis	Number of procedures	Percentage of sample
**Peptic ulcer disease**	90	29.9
**GAVE**	77	25.6
**Angiodysplasia**	40	13.3
**Esophageal varices**	37	12.3
**Gastritis**	14	4.6
**Dieulafoy lesion**	9	3.0
**Esophageal ulcer**	5	1.7
**Esophagitis**	5	1.7
**Gastric varices**	4	1.3
**Mallory-Weiss tear**	3	1.0
**Carcinoma**	3	1.0
**Other** ^a^	14	4.6
**Total**	301	100.0

Abbreviation: GAVE, gastric antral vascular ectasia.

a Other includes: Gastrojejunostomy bleed, HIT, and not specified.

The rate of PPI deprescription among patients from group 1 (2015-2018) was significantly lower than that of group 2 (2019-2022) (4% vs 15%: *P *< .002) (Table 3). This trend was consistent among the 249 unique patients who were documented to have a first-time endoscopic intervention (excluding cases with repeat endoscopic treatment). When repeat visits were excluded group 1 had a significantly lower PPI deprescription rate than group 2 (3.8% vs 16.4%; *P *< .002). The most common type of deprescription event was complete PPI deprescription with a rate of 58% (Table 4). In addition, among patients with multiple UGIB that were treated in our study 10% (5/52) were deprescribed their PPI. The median time interval between PPI deprescription and presentation for endoscopy in the study was 12.3 months.

**Table 3.** Chi-squared analysis of proton pump inhibitor (PPI) deprescription across time.

	Group 1	Group 2	Total
**PPI deprescribed**	5	28	33
**PPI non-deprescribed**	113	155	268
**Total**	118	183	301

Fisher exact test: *P *< .002.

**Table 4.** Characterizing type of proton pump inhibitor deprescription events in the cohort.

	Group 1 events	Group 2 events	Total number of events	Percentage
**Complete deprescription**	3	16	19	57.6
**Frequency reduction**	1	10	11	33.3
**Dose reduction**	1	2	3	9.1
**Total**	5	28	33	100.0

**Table 5.** Proton pump inhibitor (PPI) deprescription excluding those with gastric antral vascular ectasia.

	Group 1	Group 2	Total
**PPI deprescribed**	3	22	25
**PPI non-deprescribed**	90	109	199
**Total**	93	131	224

Fischer exact test: *P ***<** .001.

### PPI deprescription excluding patients with GAVE

We performed a subgroup analysis excluding those with an endoscopic diagnosis of GAVE (Table 5). The rationale for exclusion is that patients with GAVE and overt gastrointestinal bleeding are less likely to benefit from PPI therapy, unlike patients with PUD. The rate of PPI deprescription among patients without GAVE in group 1 (2015-2018) was significantly lower than group 2 (3% vs 17%; *P *< .001).

### PUD subgroup analysis

As part of our subgroup analysis, we analyzed the PPI deprescription rate among patients with PUD. In group 1 (2015-2018) there were no PPI deprescription among the 42 patients. Meanwhile, in group 2 (2019-2022) there was a significantly higher PPI deprescription rate (0% vs 16%; *P *= .028) (Table 6). Among 90 patients with PUD, 88 were documented as a first-time endoscopic therapy, while 2 were documented as a repeat endoscopic treatment.

**Table 6.** Proton pump inhibitor (PPI) deprescription among patients with peptic ulcer disease.

	Group 1	Group 2	Total
**PPI deprescribed**	0	6	6
**PPI non-deprescribed**	42	42	84
**Total**	42	48	90

Fischer exact test: *P ***=** .028.

We analyzed the proportion of patients who were prescribed an anticoagulant or antiplatelet medication at the time of endoscopic intervention. The prevalence of such therapy was lower among patients who underwent PPI deprescription compared to those who did not, but this difference was not statistically significant (33% vs 50%; *P *= .096) (Table 7).

**Table 7.** Antiplatelet/anticoagulant use by proton pump inhibitor (PPI) deprescription status.

	Antiplatelet/anticoagulant	No antiplatelet/anticoagulant	Total
**PPI deprescribed**	11	22	33
**PPI non-deprescribed**	134	134	268
**Total**	145	156	301

Fisher exact test: *P *= .096.

## Discussion

The findings of our study demonstrate an increased rate of PPI deprescription among patients with UGIB in recent years. These findings align with our previous study, which showed a similar trend in PPI deprescription among patients with esophageal strictures.^7^ Our cohort represented a heterogeneous group of patients with conditions ranging from GAVE to Mallory-Weiss tears. To control for this variability, we performed a subgroup analysis focusing on patients with PUD, for whom PPI deprescription would be more likely to be causative for the presenting bleed. Within this subgroup, the trends were consistent with the larger cohort. Notably, there was no PPI deprescription during the first time period, while a significantly higher rate of deprescription was observed during in the later period for patient with PUD.

Given the heterogeneity of our patient cohort and the high proportion of patients with GAVE, we conducted a subgroup analysis excluding these patients (Table 6). Our analysis further confirmed the significantly higher rates of PPI deprescription in the second time period compared to the first.

To control for potential confounding effect of medications, we recorded antiplatelet and anticoagulant medications at the time of endoscopic therapy. While the prevalence of these medications was lower among the deprescribed patients, there was no statistically significant difference between patients who were deprescribed their PPIs and those who were not.

As outlined in the Methods section, the dates for the 2 groups were selected based on dissemination of PPI deprescription guidelines to physicians in Newfoundland and Labrador. The observed differences in PPI deprescription between the 2 groups may reflect physicians and primary care providers applying these guidelines to patients in the general population. The *Canadian Family Physician* journal published clinical guidelines during this period, recommending that PPI deprescription guidelines not be applied to “those who have Barrett’s esophagus, severe esophagitis grade C or D, or with documented history of bleeding gastrointestinal ulcers.”^8^ This is particularly important as we identified cases of patients with PUD who were deprescribed their PPI after these recommendations were published. This raises the possibility of inappropriate application of PPI deprescription guidelines. Furthermore, the American Gastroenterological Association has reinforced these principles in a recent clinical practice update which states: “Patients at high risk for upper gastrointestinal bleeding should not be considered for PPI de-prescribing.”^9^ In our study, we observed multiple instances of patients presenting with repeat UGIB who were deprescribed their PPI.

There are other possible explanations beyond PPI deprescription guidelines that may have contributed to the higher rate of PPI deprescription in the second time period. These factors could include reduced access to family physicians and primary care providers, as well as barriers to care imposed by the COVID-19 pandemic.

This study has several limitations. The patient sample was small and collected from 2 academic gastroenterology practices at a single centre. The retrospective study design limited our ability to collect all data points from each patient. Our cohort was heterogeneous, including patients with UGIB ranging from GAVE to esophageal varices. We acknowledge that patient with GAVE have different disease characteristics compared to other patients, such as those with PUD. As such, patients with GAVE may not respond completely to PPI medications and may be difficult to treat endoscopically. Therefore, we performed a subgroup analysis excluding them to validate the trends of the overall cohort.

The study did not have access to pharmacy dispensing data and we were unable to classify the proportion of deprescription events that were patient driven (self-deprescription) as opposed to physician instructed. We were unable to document adverse outcomes attributable to inappropriate deprescription. As all research subjects were recruited in Newfoundland, Canada, there are unique factors to the patient population and healthcare centre that may limit the generalizability of our findings to other regions.

In conclusion, we found that more patients receiving endoscopic therapy for UGIB in 2019-2022 had their PPI deprescribed than in 2015-2018. We identified numerous cases of PPI deprescription among patients with repeat UGIB. These findings are consistent with our previous research on patients with esophageal strictures. It is possible that PPI deprescription guidelines released during this period played a role in these observations. Appropriate application of PPI guidelines and the continuation of PPIs for patients with strong indications are important for patient care. As a result of feedback from clinicians, including those involved in this study, Choosing Wisely Canada will publish a revised PPI deprescription toolkit with more explicit guidance on which groups of patients should not be considered for deprescription.^10^ This document was published in May, 2025.^10^ Further research involving larger, multicentre patient cohorts can help corroborate our findings.

## Supplementary Material

gwaf021_Supplementary_Data

## Data Availability

The data underlying this article will be shared on reasonable request to the corresponding author.
